# Changes in Physician Work Hours and Patterns During the COVID-19 Pandemic

**DOI:** 10.1001/jamanetworkopen.2021.14386

**Published:** 2021-06-23

**Authors:** Xiaochu Hu, Michael J. Dill

**Affiliations:** 1 Association of American Medical Colleges, Washington, DC

## Abstract

This study examines the hours worked and patterns of work activities before and during the COVID-19 pandemic among US physicians.

## Introduction

The COVID-19 pandemic has been associated with loss of revenue, reduced work hours, and reduced earnings for physicians in the United States.^1,2,3^ Furthermore, pandemic restrictions and related regulatory changes allowing physicians greater flexibilities potentially altered physicians’ work activities and environments.^4,5^ We analyzed a longitudinal data set to examine changes in US physician work hours and activities before and after the COVID-19 pandemic emerged.

## Methods

We analyzed the Current Population Survey (CPS) basic monthly data from January 2019 to December 2020, obtained through the Integrated Public Use Microdata Series (IPUMS). Administered by the US Bureau of the Census, the CPS contains demographic and employment information. The response rate ranged between 65% and 83%. (See eAppendix 1 in the Supplement for detailed information about the data.) Participants were interviewed 8 times during a 16-month period, allowing for longitudinal analyses.

This study followed the Strengthening the Reporting of Observational Studies in Epidemiology (STROBE) reporting guideline. This study was deemed exempt from review by the American Institute for Research institutional review board because it used publicly available survey data and was not human subjects research in accordance with 45 CFR §46. Participants gave consent prior to each interview.

First, we estimated panel regression models with individual- and time- fixed effects (year and month) using linked person-level data. This controlled for time-invariant factors (eg, sex, race/ethnicity, medical specialty) and minimized potential bias due to a decrease in survey response rate. Time-invariant factors, whether available in the survey (eg, sex, race/ethnicity) or not (medical specialty), were not included in the statistical model as independent variables. Next, we used pooled data to compare pre–COVID-19 (January 2019 to March 2020) and during COVID-19 (April to December 2020) mean percentages of physicians working full-time, performing the same activities, and laid off. Additionally, we compared mean percentages of parents of preschool-aged children (younger than 5 years of age) among both male and female physicians. Survey weights were applied in all analyses, and regression models were clustered at the person level. Comparisons were tested with simple linear regression, and *P* < .10 was considered statistically significant. Analyses were performed using Stata version 14.1 (StataCorp) in February 2021.

## Results

Our data set included 8853 observations of 2563 unique physicians (weighted demographic data were 5311 male physicians [60%] and 6230 White physicians [70.4%]; weighted mean [SD] age was 46.7 [13.4] years). In January 2019, mean weekly hours worked per week by physicians was 50.8 (95% CI, 47.7-51.8 hours per week). In March 2020, this began to decrease (49.2 hours per week; 95% CI, 48.0-50.3 hours per week; *P* = .05) and reached the lowest point in May 2020 (47.5 hours per week; 95% CI, 46.1-48.8 hours per week; *P* < .001) (Figure). Work hours stabilized in the summer before reaching another low in November. Mean weekly work hours in December 2020 were 47.8 (95% CI, 46.6-49.1 hours per week; *P* < .001), an approximate 6% decrease from January 2019.

**Figure.**
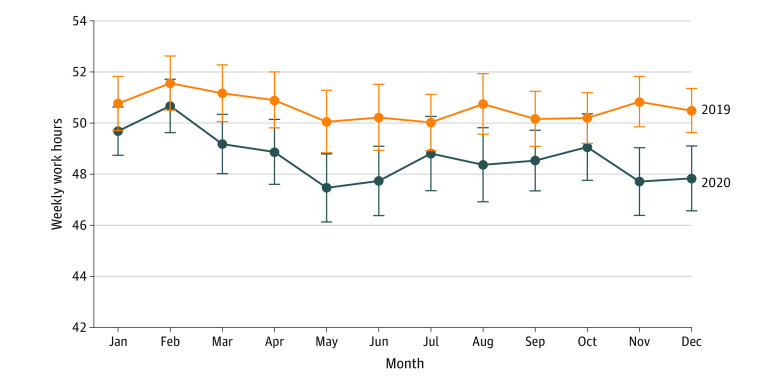
Physicians’ Mean Weekly Work Hours, January 2019 to December 2020 The authors’ calculations were based on the Integrated Public Use Microdata Series Current Population Survey basic monthly data from January 2019 to December 2020. *P* values < .10 for July and October 2020. *P* values < .05 for March, April, August, and September 2020. *P* values < .01 for May, June, November, and December 2020. Error bars denote 95% CIs, which accounted for the use of sampling weights and for clustering at the individual level. See eAppendix 2 in the Supplement for additional notes.

The percentage of physicians reporting full-time work status declined from 84.17% (95% CI, 83.13% to 85.22%) before COVID-19 to 80.65% (95% CI, 79.03%-82.27%) (*P* < .001) during the pandemic, and those who claimed to “still have the same activities” at work declined from 83.90% (95% CI, 82.65% to 85.14%) to 78.00% (95% CI, 76.01% to 79.98%) (*P* < .001) (Table). Although rare, the percentage of physicians reporting being laid off increased from 0.05% (95% CI, −0.01% to 0.11%) to 0.45% (95% CI, 0.21% to 0.70%) during COVID-19 (*P* < .001). The percentage of parents of preschool-aged children among full-time, female physicians decreased from 17.98% (95% CI, 16.05% to 19.91%) to 14.10% (11.59% to 16.60%) (*P* = .009) and did not significantly change among male physicians.

**Table.** Comparison of Work Status and Activities Before and During the COVID-19 Pandemic^a^

Characteristic	Unweighted sample size, No.	Mean (95% CI), %		*P* value
		Pre-COVID	During-COVID	
Full-time	8853	84.17 (83.13 to 85.22)	80.65 (79.03 to 82.27)	<.001
Still have the same activities	6762	83.90 (82.65 to 85.14)	78.00 (76.01 to 79.98)	<.001
Laid off	8853	0.05 (−0.01 to 0.11)	0.45 (0.21 to 0.70)	<.001
Full-time, female physicians with children aged <5 y^b^	2743	17.98 (16.05 to 19.91)	14.10 (11.59 to 16.60)	.009
Full-time, male physicians with children aged <5 y^c^	4561	16.17 (14.71 to 17.63)	17.22 (14.98 to 19.46)	.36

^a^ The authors’ calculations were based on the IPUMS CPS basic monthly data January 2019 to December 2020. See eAppendix 3 in the Supplement for additional notes.

^b^ Among female, full-time physicians.

^c^ Among male, full-time physicians.

## Discussion

This study found that physicians’ work hours have significantly decreased since the start of the COVID-19 pandemic in the US, accelerating an existing, gradual decline.^6^ There was also a decreased percentage of physicians working full-time, a rise in the percentage who were laid off, and increased changes in physicians’ usual activities. These observed changes may reflect the decrease in health care utilization^1^ and the increased flexibilities instigated by COVID-19–driven regulations.^4^ The decline in the percentage of parents with preschool-aged children among only female physicians may suggest a disproportionate uptake of childcare responsibilities among female physicians.

This study leveraged the timely, longitudinal design of CPS data to provide early evidence of changes in physician work hours and patterns. However, this study also had limitations: the CPS sample size of physicians was small and lacked detailed information. Future studies analyzing physician-specific data are needed to fully examine the impact of the pandemic on the physician workforce.

## Notes

Supplement. **eAppendix 1.** IPUMS CPS and CPS Response Rates **eAppendix 2.** Additional Notes for Figure **eAppendix 3.** Additional Notes for Table

## References

[1] Rubin R. COVID-19's crushing effects on medical practices, some of which might not survive. JAMA. 2020;324(4):321-323. doi:10.1001/jama.2020.11254 32556122

[2] American Academy of Family Physicians. AAFP NRN/Graham Center surveys track COVID-19’s effects. Accessed October 22, 2020. https://www.aafp.org/news/practice-professional-issues/20200529covidsurveys.html

[3] Brubaker L. Women physicians and the COVID-19 pandemic. JAMA. 2020;324(9):835-836. doi:10.1001/jama.2020.14797 32735329

[4] Center for Medicare and Medicaid Services. 2021. Physicians and other clinicians: CMS flexibilities to fight COVID-19. Accessed May 25, 2021. https://www.endocrine.org/-/media/endocrine/files/membership/cms--covid-phys-fact-sheet.pdf

[5] Hu X, Dill JM, Haberman M, Impact of COVID-19 on physician workforce: an examination of the staffing strategies and concerns of faculty practice plans. Association of American Medical Colleges. Published 2020. Accessed November 16, 2020. https://www.aamc.org/media/49001/download

[6] Staiger DO, Auerbach DI, Buerhaus PI. Trends in the work hours of physicians in the United States. JAMA. 2010;303(8):747-753. doi:10.1001/jama.2010.168 20179284 PMC2915438

