# Supplementary material for: Changes in Physician Work Hours and Patterns During the COVID-19 Pandemic
Source: JAMA Netw Open. 2021 Jun 23;4(6):e2114386. doi: 10.1001/jamanetworkopen.2021.14386 (PMC8223097; doi:10.1001/jamanetworkopen.2021.14386)
Supplement: Supplement. — eAppendix 1. IPUMS CPS and CPS Response Rates eAppendix 2. Additional Notes for Figure eAppendix 3. Additional Notes for Table [file jamanetwopen-e2114386-s001.pdf]

Supplemental Online Content

Hu X, Dill MJ. Changes in physician work hours and patterns during the COVID-19 pandemic. *JAMA Netw Open.* 2021;4(6):e2114386.
doi:10.1001/jamanetworkopen.2021.14386

eAppendix 1. IPUMS CPS and CPS Response Rates

eAppendix 2. Additional Notes for Figure

eAppendix 3. Additional Notes for Table

This supplemental material has been provided by the authors to give readers additional information about their work.

© 2021 Hu X et al. *JAMA Network Open.*

eAppendix 1. IPUMS CPS and CPS Response Rates

Source for obtaining IPUMS CPS data: Sarah Flood, Miriam King, Renae Rodgers, Steven Ruggles and J. Robert Warren. Integrated Public Use Microdata Series, Current Population Survey: Version 8.0 [dataset]. Minneapolis, MN: IPUMS, 2020. https://doi.org/10.18128/D030.V8.0**.** Accessed February 4, 2021.

Data used in this analysis included individual whose occupations are reported as "physicians and surgeons", including residents. Informed consent was obtained prior to the interviews by the US Census Bureau, who conducted the survey. The CPS typically has very high response rates; however, response rates declined during the pandemic due, in part, to regulations impacting the data collection process (e.g. call centers having to close; inability to conduct in-person interviews). More detailed documentation on the effects of the pandemic on data collection can be found here IPUMS CPS. The panel regression model used in this study allowed us to compare the same individuals pre- and during-COVID-19, and therefore minimized potential bias resulting from the decline in response rates.

eAppendix 2. Additional Notes for Figure

Only physicians reporting weekly hours between 21-99 are included, reducing the N to 7,758 observations/2,345 unique individuals. Estimates derived from individual- and time-fixed-effect panel regression analysis. All analyses accounted for use of sampling weights and clustering of observations within individuals.

© 2021 Hu X et al. *JAMA Network Open.*

eAppendix 3. Additional Notes for Table

Since Stata does not support *t* test with weight, the 95% CI were estimated by means comparison accounting for use of sampling weights. P-values generated by simple regression. All analyses accounted for use of sampling weights. "Full time" is defined as the IPUMS CPS variable "WKSTAT" = 11 (Full-time hours (35+), usually full-time). The IPUMS CPS variable "Still have the same activities" indicates whether the respondent's usual work activities or duties have changed during the previous month. Not available to persons in their 1st and 5th months.

© 2021 Hu X et al. *JAMA Network Open.*
